# Seeing ourselves in anatomy: Embedding equity, diversity, and inclusivity to transform learning in a UK BSc curriculum

**DOI:** 10.1002/ase.70333

**Published:** 2026-08-27

**Authors:** Michelle Welsh, Ourania Varsou

**Affiliations:** ^1^ Anatomy Facility, School of Medicine, Dentistry and Nursing, College of Medical, Veterinary and Life Sciences University of Glasgow Glasgow UK

**Keywords:** anatomical education, equality, diversity and inclusion, equity, diversity and inclusion, intersectionality, resource diversity, scaffolded constructivist pedagogy

## Abstract

Embedding intersectional equity, diversity, and inclusion (EDI) in anatomy education is essential to reflect the growing diversity of student populations, support all learners, and prepare graduates for a more inclusive, socially responsive global workforce. Anatomical textbooks and models have typically focused on white, cis‐gender, able‐bodied individuals who perpetuates biases and underrepresentation, impacting learners' professional development. We embedded five workshops in the junior honors BSc anatomy curriculum, which all adopted an intersectional EDI approach. These were strongly anchored to anatomy as a discipline and framed around learners building knowledge through social interaction, encouraging active student engagement and reflection. A mixed methods analysis using pre‐ and postteaching surveys and assessment data from 2021 to 2025 showed that the anatomy‐focused EDI content challenged students to explore personal and disciplinary representation, bias, and diversity through real‐world examples such as textbook imagery, donor demographics, and historical figures. By combining collaborative tasks, critical reflection, and authentic assessments, students developed cultural awareness, and personal accountability, embedding inclusive thinking into both their academic practice and professional identity, with their understanding shifting over time from more basic awareness to reflective, critical engagement. Students also consistently achieved higher grades in EDI coursework and exam essays compared to non‐EDI assessments. This approach allowed students to build complexity across the sessions, from historical critique to structural analysis and applied outputs. These findings support the continued integration of EDI as a dynamic and essential component of anatomy and broader biomedical curricula while also offering a transferable framework for other academics to adopt.

## INTRODUCTION

All learners, irrespective of their identities and demographics, should have equitable access to education and feel represented and included in the learning experience offered, not least because this sense of belonging has been shown to be central to student success, particularly for those from underrepresented or nontraditional backgrounds.^1^ Students who feel represented and supported in their studies report greater engagement, motivation, and confidence, known predictors of academic performance.^2^ This is increasingly important as we recognize the growing diversity of student populations; for example, the proportion of UK‐domiciled full‐time students from racially minoritized backgrounds entering full‐time higher education in England increased from 24% in 2010 to 35% in 2023.^3^ According to the *British Medical Journal*, this diversity is also evident within undergraduate medicine with 31% of the 16,000 students at UK medical schools identifying as from a minoritize ethnicity, with 10% Indian ethnicity, 3% Black, 5% Pakistani, and 4% mixed ethnicity.^4^ Importantly, our student body is not only racially diverse but also reflects a wide range of other characteristics and demographics. For instance, the proportion of UK‐domiciled full‐time students reporting a disability has doubled since 2010, reaching 19% in 2021.^5^ Therefore, when embedding equity, diversity, and inclusion (EDI) into higher education curricula, it is important to consider intersectionality, which recognizes that individuals experience overlapping systems of oppression and privilege shaped by multiple aspects of identity such as race, gender, sexuality, social background, and ability.^6^ While many researchers have undertaken important research around decolonizing the anatomy curricula,^7^ institutions have also started to move beyond this to consider instead a more intersectional approach to foster greater inclusion for students.^8^, ^9^ Rather than treating each identity in isolation, intersectionality considers how these factors interact to create unique challenges for learners and applying intersectionality can help to break down siloes and promote a more nuanced understanding of EDI.

A lack of representation within curricular content can impact the students' academic achievements as well as their sense of belonging and attitude toward learning^10^ therefore considering EDI within higher education is no longer an optional endeavor but a pedagogical necessity requiring proactive, intentional integration across curricula. This urgency arises not only from the ethical imperative to create inclusive academic spaces and learning environments,^11^ but also from the practical need to equip our graduates for an increasingly globalized and culturally diverse job market and society.^12^, ^13^ The UK Quality Assurance Agency for Higher Education (QAA) biosciences benchmark and the US Council for Higher Education Accreditation (CHEA) both advocate for the proactive integration of EDI across curricula,^14^, ^15^ with the hope that embedding EDI into educational practices and curriculum design can also contribute to improving student retention and reducing the degree awarding gap.^1^, ^2^, ^16^, ^17^, ^18^ This gap, sometimes called the attainment or degree classification gap, refers to measurable differences in the proportion of students from different demographic groups who graduate with higher‐level degree classifications, even when factors like prior attainment or socioeconomic status are controlled for. When students feel excluded or undervalued in learning materials or environments, it can undermine engagement and sense of belonging, known contributors to differential achievement.^2^, ^17^, ^18^ This is likely to contribute to differential academic outcomes and awarding gaps therefore active strategies are required to support all students to feel included and meet their potential. For example, despite the increased representation of Black students in Higher Education in the United Kingdom, only 17% of Black students achieved a first‐class degree in 2023 compared to 36% of white students.^12^, ^13^, ^19^

It is also important to acknowledge the influence of the hidden curriculum in shaping students' experiences and understandings of the discipline. The hidden curriculum encompasses the unspoken norms, values, and messages that students absorb through their learning environment,^20^ often without explicit instruction. In anatomy, this includes the notion of the “default body,” reinforced through the historical predominance of white, cis‐male, able‐bodied figures in anatomical texts, and models^21^; gaps in representation across race, sex, age, and ability^2^; and the privileging of particular types of language and imagery that perpetuate androcentrism and normative assumptions.^22^ It also extends to the implicit messages communicated through body donor demographics and donation practices, which tend to overrepresent older white donors,^23^ as well as limited visibility of disabled bodies and accessibility considerations within traditional anatomy curricula.^24^ Together, these hidden norms can unintentionally reinforce narrow or exclusionary understandings of human variation, shaping professional identity formation in ways that privilege certain bodies and experiences over others.^25^ Recognizing these hidden influences provides important context for the present study, as it highlights why explicit, structured engagement with EDI principles is necessary to challenge entrenched norms and support more inclusive, reflexive learning.

As well as the importance of supporting our diverse student population, it is also vital to recognize that graduates are no longer expected to possess only knowledge and technical expertise; they must also demonstrate transferable attributes like empathy, communication, adaptability, and cultural competence, the ability to navigate complex interpersonal dynamics and a nuanced understanding of diverse lived experiences.^26^ This is increasingly true for students in biomedical and health‐related disciplines such as human anatomy, with research demonstrating that EDI‐related interventions improved professionalism of healthcare practitioners.^24^, ^27^ For example, research demonstrated that teaching the concept of gender within anatomy and physiology courses positively impacted future healthcare practitioners' interactions with individuals different from themselves such as transgender or nonbinary individuals.^28^ Furthermore, previous research demonstrates a need for a more intentional, iterative, and multifaceted approach to embedding EDI in medical education,^8^ suggesting that such changes are only truly effective if they are meaningful and sustained at institutional and individual levels.^7^ Failing to prepare students for the complex sociopolitical, cultural, and biological diversity that characterizes real‐world populations has contributed to health disparities and poorer outcomes for patients based on their demographics.^26^, ^28^, ^29^

Anatomy as a discipline offers an excellent opportunity to address equity and diversity within the curriculum as many protected characteristics relate directly to the human body like aging, sex, race, and disability. Yet despite this, the discipline continues to face challenges in this area; many anatomy educators reported feeling unsure where to start and concerned about constraints when engaging with EDI,^7^, ^8^, ^9^, ^28^ limited further by traditional anatomical sciences resources predominantly representing only a white, slim, cisgender, able‐bodied male or female body, often without visible pathologies, disabilities, or anatomical variation.^21^, ^23^, ^30^, ^31^, ^32^ Anatomists have recognized the importance of making our discipline more inclusive and representative of the community we educate but also treat in healthcare practices.^7^, ^28^ This representation extends beyond visual anatomical imagery; for example, the continued use of eponymous terms and noninclusive language can perpetuate stereotypes.^33^ Many of these terms already have more accurate descriptive alternatives that do not increase students' cognitive load (e.g., Graffian follicle vs. tertiary follicle).^34^ This narrow portrayal has long been upheld as the standard or “norm” in anatomy, reflecting the historical biases.^7^ However, anatomy students view inclusivity in teaching as a shared value and wish to see greater diversity reflected in their learning resources.^2^, ^8^ Lack of representation in anatomical teaching has tangible implications for student engagement and sense of belonging, impacting their academic confidence, achievement, and professional identity formation, particularly for students from minoritized backgrounds.^35^, ^36^

Teaching anatomy through an intersectional lens offers the opportunity to humanize the subject and deepen students' understanding of how diverse characteristics and structural inequalities are embedded in research practices and healthcare settings. Without this intersectional and critically reflexive perspective, we risk perpetuating cycles of exclusion that disadvantage learners and ultimately undermine efforts toward equitable healthcare delivery and inclusive research practice.^7^ Through this study, the authors aimed to apply this approach to diversifying the BSc Honors in Anatomy program at the University of Glasgow via the development and embedding of a series of bespoke EDI workshops into year 3 of the degree and investigate the impact of this initiative on the student experience and their learning. In doing so, the authors aimed to demonstrate this as a scalable model that could be adapted across disciplines, institutions and sectors to meaningfully integrate intersectional EDI in ways that enhance belonging, critical thinking, and academic success.

To strengthen the theoretical framing of this study, it is useful to clarify how we define EDI within the context of anatomical education. Diversity refers to recognizing the broad spectrum of human difference, including characteristics such as race, sex, gender, age, disability, and social background, while equity concerns addressing the structural and systemic factors that influence fairness, access, and opportunity in educational settings (28{Ludwig, 2020 #445}). Inclusion moves beyond representation to focus on cultivating learning environments where all students feel valued, respected, and able to participate fully, thereby fostering a genuine sense of belonging.^2^ Together, EDI operates as an overarching framework for policies and practices that promote fair treatment, equal opportunity, and protection from discrimination.^28^ We further situate diversity using the Dimensions of Human Diversity, articulated in the Diversity Wheel proposed by Loden and Rosener and later adapted by the Cultural Competence Learning Institute, which conceptualizes diversity as multilayered—encompassing innate personal attributes, acquired identities, and wider organizational or societal contexts. This framework underscores that diversity in anatomy extends far beyond visible physical traits, highlighting the ways intersecting identities shape learners' experiences, representation, and engagement with anatomical sciences. In parallel, decolonization within anatomy involves interrogating and dismantling the historical privileging of white, Western, cis‐male bodies, and perspectives in anatomical resources, donor demographics, and disciplinary narratives.^30^, ^37^ By embedding EDI‐informed practices—such as diversifying teaching materials, critically examining donor representation and challenging historical bias—this study contributes to wider efforts to decolonize the anatomy curriculum and reposition the discipline as more inclusive, representative, and socially accountable.^7^

## METHODS

### Positionality

Michelle Welsh (she/her) is a first‐generation academic with a BSc (Hons) in Anatomy and a PhD, and over 15 years' experience teaching anatomy in higher education. She is UK‐born, White, cis‐gendered, and able‐bodied and recognizes that these identities confer privilege and influence within academic spaces. Her approach to anatomy education is grounded in a commitment to EDI, challenging normative representations of the body and foregrounding the ethical dimensions of anatomical knowledge. She adopts a reflexive practice, recognizing positionality as dynamic and responsive to lived experience and systemic inequities.

Ourania Varsou (she/her) is a Greek‐born, UK‐based first‐generation PhD holder whose positionality is shaped by migration, social mobility, and long‐term engagement with UK academic and professional systems. As a medically qualified senior academic with interdisciplinary and transdisciplinary experience across higher education, healthcare, the private sector, and nonprofit organizations, she occupies positions of both expertise and influence. She recognizes the power and responsibility that accompany this positioning, particularly in relation to the use of emerging technologies in education and healthcare and approaches her work with ongoing critical self‐reflection, ethical responsibility, and a commitment to co‐creating knowledge that is inclusive, socially accountable and attentive to governance, equity, and impact.

Articulating our positionality is essential in this study because our identities and professional locations shape how we interpret issues of power, representation, and inclusion in anatomical education and making these influences explicit strengthens the reflexivity and ethical integrity of the research. Furthermore, acknowledging our positionality also clarifies how our roles as educators may influence students' willingness to share perspectives and engage with sensitive EDI content, helping us to critically examine the power dynamics inherent in data collection and to ensure that students' voices are represented ethically, respectfully, and without coercion.

### Pedagogical framework: Universal design for learning and scaffolded constructivist pedagogy

The educational frameworks underpinning our EDI intervention are grounded in universal design for learning (UDL) and scaffolded social constructivist pedagogy. UDL recognizes learner diversity as a strength and aims to make learning accessible, inclusive, and effective for all learners by proactively designing teaching, learning activities, and assessments to accommodate learner variability from the outset, rather than retrofitting adjustments later.^38^ By offering multiple ways to engage with, access, and demonstrate learning, UDL creates inclusive environments that support student success and equity across higher education. Scaffolded social constructivist pedagogy blends two powerful educational theories: social constructivism and instructional scaffolding, whereby learning is viewed as an active, student‐centered process attained via collaborative interactions, with students taking increasing responsibility and ownership of the learning process over time. Our learning model scaffolded students' knowledge and engagement starting with introductory topics anchored on familiar anatomical topics and gradually moving to more complex and challenging EDI content, designing each session to be accessible and consider the diverse needs of learners.^32^ This approach placed equal emphasis on theoretical knowledge acquisition along with the social interactions that supported learning and personal development. This combination led to discussions not only focused on anatomical disciplinary knowledge but also wider academic and societal EDI topics resulting in greater awareness and personal reflection. In our approach, the content was not presented as an “add‐on,” but rather meaningfully integrated within core anatomical topics, reinforcing its relevance to disciplinary learning and avoiding a tokenistic approach which was a central motivator for engagement with content which could be seen by students as supplementary. Another key component was that students were not passive recipients, but active contributors invited to select their own topics and bring their prior academic lived experiences along with their thoughts and perspectives into the learning process.^39^ This allowed for a deeper, more personal and authentic engagement with the themes, enhancing learners' sense of belonging.^40^ Finally, the sessions were designed through an intersectional lens, encouraging students to recognize the impact of overlapping identities and understand how social identities and systemic inequalities shape both the practice and experience of anatomical education and healthcare.

To support this pedagogical framework, we established a safe, inclusive learning environment aligned with the recommendations of the UK QAA biosciences benchmark^14^ which encouraged active participation, mutual respect and individual responsibility for fostering inclusive practices. A code of conduct (Figure S1) ensured that respectful dialogue, inclusive language and openness to sensitive discussions were prioritized throughout. This code of conduct goes beyond the protected characteristics listed in the UK Equality Act 2010, which provides an essential legal framework for protecting individuals with specified protected characteristics. The UK Equality Act is compliance‐driven which can encourage a narrow approach to EDI therefore the code of conduct aims to expand this to also consider intersectionality, structural inequities, and broader dimensions of inclusion within educational practice. Workshops were discussion‐based designed to be low‐stakes, fostering the element of psychological safety and empowering everyone to talk openly and honestly, challenge assumptions, and learn reciprocally from one another with the aim of reaching own personal and academic self‐actualization in EDI.

### BSc in Anatomy (Honors) Program

The authors embedded EDI workshops into year three of the BSc in Anatomy (Honors) Program at the University of Glasgow, which is a 4‐year undergraduate degree offering students a comprehensive foundation in life sciences with a focus on human anatomy. The program is designed to prepare graduates for a wide range of careers in biomedical research, healthcare professions, education, digital technologies, other allied healthcare sectors, and beyond. As well as developing discipline‐specific skills and knowledge, the program also fosters nondiscipline‐specific skills crucial for employability, such as communication, teamwork, professionalism and independent learning through group projects, presentations, peer collaboration, and research‐led assignments,^41^ aligning with the University's commitment to producing well‐rounded, resourceful and investigative graduates (UofG Graduate Attributes). Approximately 40 students per year enroll in the BSc in Anatomy (Honors) Program, with data since 2015 showing that the majority are female (82%), white (83%), UK resident (87%), full‐time, studying their first degree, and with no known disability (84%).

In years 1 and 2 (prehonors), students study a broad‐based life sciences curriculum that includes fundamentals in biomolecular and biomedical sciences as well as core skills like statistics, science communication, and data analysis and interpretation. In year 2, students also learn more about the UK Equality Act 2010^42^ and why equality, equity, and diversity are important in academia and the life sciences, how the law protects people from unfair treatment and how to be an active bystander with the view to our graduates contributing to reducing the occurrence and acceptance of discriminating or harassing behaviors in the sector.^43^ Following these broad pre‐honors 2 years, students formally enter the BSc Anatomy program in year 3, a 1‐year full‐time course focused on immersive anatomical study, engaging in authentic dissection‐based learning and developing discipline‐specific technical skills. Students in year 3 Anatomy also hone their specialized anatomical knowledge through lectures and workshops, deepening their understanding of research methods, critical thinking, academic writing, and statistics (course catalogs 3A and 3B). Critical reflection of this course in 2021 by the authors in their roles as course coordinators identified that students were being taught anatomy through a very traditional lens, focusing on the anatomy of able‐bodied white European males, which resulted in a lack of diversity, representation, and inclusion in this course. In this research report, the authors have discussed a series of EDI workshops which were developed and integrated into year 3 of the BSc program, before students progressed into their research focused final year (Figure 1).

**FIGURE 1:**
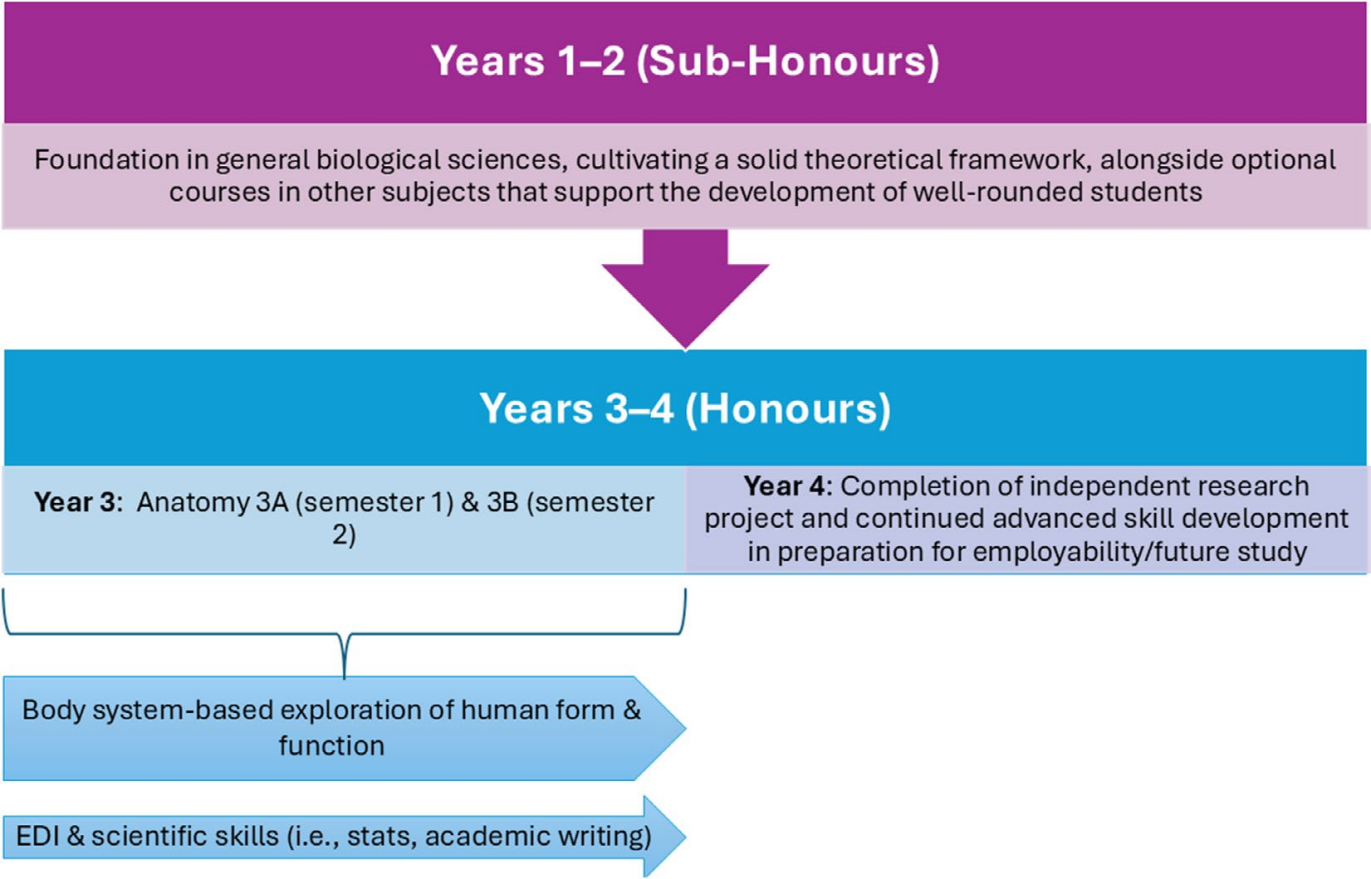
Structure of the anatomy BSc with the EDI workshops highlighted in yellow for year 3.

### Thematic anchors: Real‐world anatomy‐grounded EDI topics

The EDI workshops embedded in year 3 anatomy were structured around three interrelated themes that contextualized EDI within anatomical sciences (Figure 2). These themes^2^ were designed to reflect the relevance of EDI to anatomists as individuals, to the diversity of the human body, and also the diversity, or lack of, within our body donation program, aiming to prompt reflection and spark deep critical thinking.^41^ Each 2‐h workshop was delivered in person by the authors, with all students in the cohort attending together. These workshops were spaced throughout the academic session with three in semester one and the remaining two in semester two.

The intended learning outcomes for these workshops were as follows:

- To explore and collaboratively discuss how inclusive and diverse past and current anatomy practices are,
- To investigate if past anatomists represent current EDI practices,
- To discuss if donors, anatomical models and illustrations represent diverse and modern anatomy,
- Investigate the diversity of the human body and pathological conditions.

**FIGURE 2:**
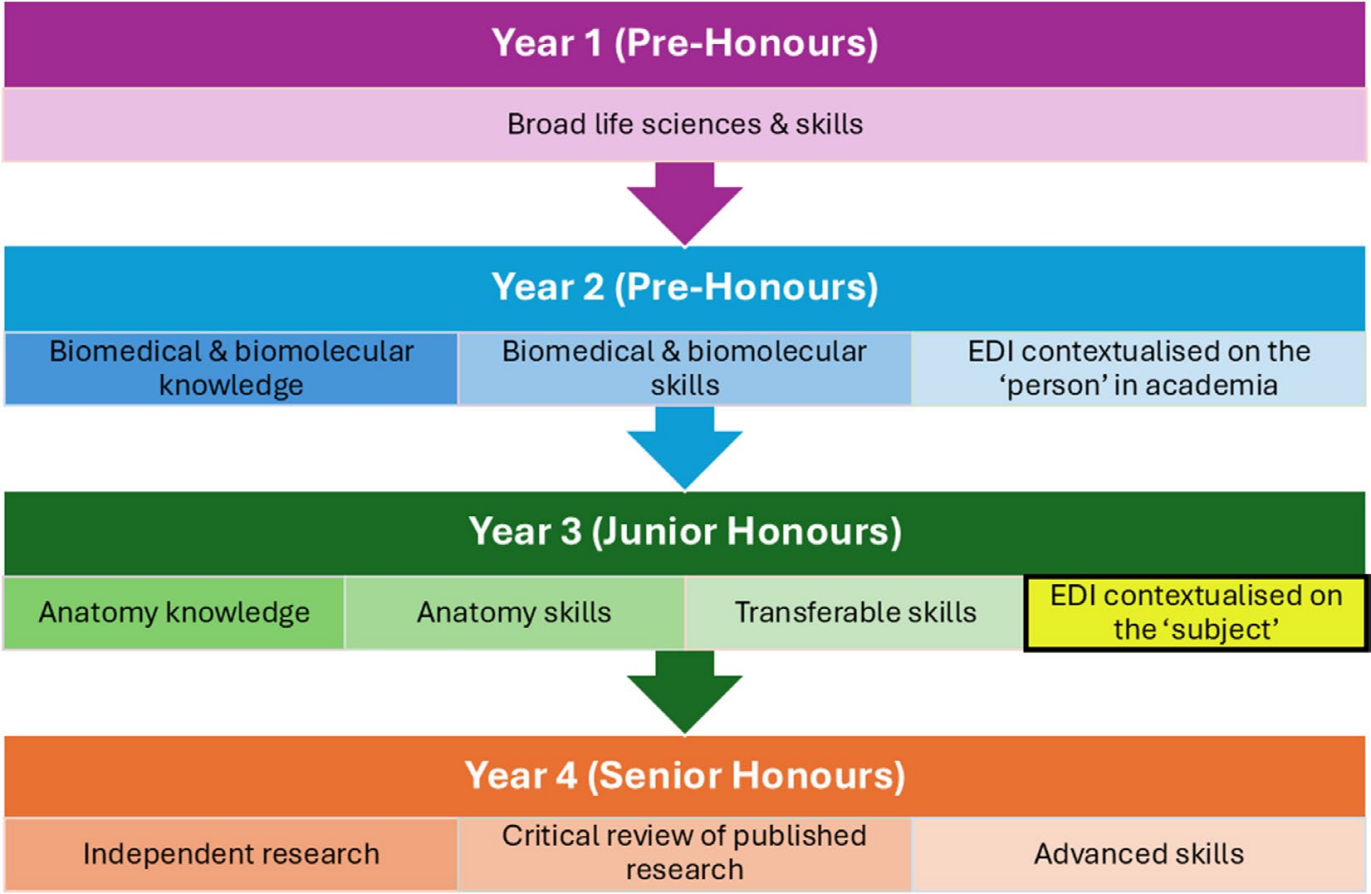
Thematic discipline‐specific anchors.

### Workshop outlines

#### Workshop 1: Introduction to EDI in anatomy

The opening EDI workshop introduced students to key EDI terminology and concepts, including the idea of the “androgenisation of anatomy,” the historical overrepresentation of male bodies in anatomical education.^31^ Students discussed this within the broader concept of androcentrism as the societal organization around masculine generics before exploring how the male body is commonly represented as the gender‐neutral standard, for example, through the dominant use of male anatomical figures to represent all sexes.^22^ This was centered around Parker et al.'s^21^ systematic visual analysis of 6044 images from anatomy textbooks published between 2008 and 2013 which demonstrated that male bodies were predominantly represented in general anatomical illustrations, while female figures were largely confined to sex‐specific sections with a lack of ethnic, age, and body type diversity and near‐total adherence to the sex binary. Through prompt questioning and use of Mentimeter (Mentimeter AB, Sweden), students were encouraged to critically reflect on whose bodies are studying and whose are missing, and how this visual framing can lead to false assumptions about the anatomy and physiology of those assigned female at birth. Such practices have real‐world implications, particularly when data or representations are not directly transferable across sexes, such as the inaccurate generalization of findings from medical research primarily conducted on male participants, thereby potentially compromising the quality and inclusivity of healthcare and education.

#### Workshop 2: Diverse anatomists

In the second workshop, students worked in small groups to research and present on a historical or contemporary anatomist who could be considered diverse rather than the classical European male anatomists who dominate history. With this in mind, students were asked to critically select at least two protected characteristics drawn from the Equality Act 2010; a UK legislation that defines nine legally protected attributes (i.e., age, disability, gender reassignment, marriage and civil partnership, pregnancy and maternity, race, religion or belief, sex and sexual orientation) that promotes fairness, equity, and prevent discrimination.^42^ Using this framework served multiple purposes: (i) it provided students with a familiar and structured starting point for exploring diversity; (ii) it aligned with nationally recognized standards in legislation; and (iii) it enabled students to make links between the legal and biological understandings of identity and inclusion without digressing into sociocultural narratives that would have been outside of the scope of this program. This activity was designed not only to encourage critical engagement with history and contributions of underrepresented anatomists but also to challenge dominant narratives in the discipline that have historically marginalized individuals based on their identities. This approach also supported the development of students' cultural competence and ethical awareness, as key attributes for our future graduates (UofG Graduate Attributes).

Although this workshop originally focused on the protected characteristics defined in the Equality Act 2010, and therefore pre‐dates the 2025 UK Supreme Court ruling that re‐centered the legal definition of “woman” on biological sex, future iterations of the session should incorporate discussion around how legal frameworks evolve and may diverge from ethical, pedagogical, and sociocultural considerations.

#### Workshop 3: Donors—Body donor representation

Anatomical donors offer a unique and irreplaceable learning tool for anatomical education,^44^, ^45^ however, little research has been done into the diversity of anatomical donors and how well they represent patient populations.^23^ This is further compounded by the underrepresentation of darker skin tones in commonly used medical and anatomy textbooks, with only 4.5% of images depicting dark skin and 21% medium.^23^, ^44^, ^45^ However, little research has been done into the diversity of anatomical donors and how well they represent patient populations.^23^ This is further compounded by the underrepresentation of darker skin tones in commonly used medical and anatomy textbooks, with only 4.5% of images depicting dark skin and 21% medium.^23^ A more equal patient and image demographic representation could enhance students' understanding of the diversity of anatomy and its clinical implications so workshop three centered on the diversity, or lack thereof, within our donors and contemporary anatomical learning resources. Discussions focused on skin anatomy as a tangible example, enabling students to contextualize variation in skin tone and how the absence of this diversity in education disproportionately affects individuals of color. Students reflected on how the demographics of donors, often older and white, can skew perceptions of what constitutes a “normal” body and as such, who was not being represented in this learning experience. These conversations allowed students to interrogate their own preconceptions while exploring the broader pedagogical implications of donor homogeneity and textbook bias. Importantly, students were asked to consider why this matters; the absence of skin tone diversity in teaching resources may hinder future clinicians' ability to recognize skin conditions across varied populations, perpetuate unconscious bias, and ultimately contribute to health inequalities. Through this workshop, students came to appreciate the responsibility of the anatomical sciences in supporting equitable research practices. Additionally, students were encouraged to recognize that contemporary donor pools in Western institutions continue to reflect historical, socioeconomic, and colonial legacies, with disproportionate representation of white, older, and socially advantaged donors. This aimed to help students situate donor diversity within wider structural contexts.

#### Workshops 4 and 5: The human body—Structures and variance

In the final two workshops, students were asked to delve deeper into how diversity manifests in human structure and disease presentation. In workshop four, students explored a disease of their choosing through a diverse lens, creating a formative wiki entry that highlighted EDI‐specific differences in disease prevalence, diagnosis, and treatment. Students reflected on the implications for research practice, particularly diagnostic accuracy, inclusivity of medical models, and the representation of patient populations. In doing so, the workshop deepened students' engagement with EDI as a discipline‐relevant aspect, moving beyond tokenism to a more integrated understanding of anatomical diversity as both a biological and social construct. In workshop five, students investigated diversity of a specific anatomical structure or body region which was linked to a summative poster assessment, empowering students to showcase their understanding of how EDI intersects with the human body; example of student work shown in Figure 3. By critically analyzing the human form through such a lens, students began to appreciate that anatomy is not a fixed or universal blueprint, but a spectrum that could also be influenced by lived experience, identity, and environment factors. In addition to the poster, students also completed a summative reflective writing task, where they articulated what they had learned from engaging with EDI throughout all five workshops. These reflections encouraged students to consider how their understanding and perspectives might have shifted and to identify concrete actions they could take to apply this learning in both academic and future professional settings. Many students developed personal action plans, outlining how they would continue to build cultural awareness, challenge bias in professional environments and advocate for inclusivity. This demonstration of heutagogy or self‐directed learning, with students taking ownership for what they will continue to learn and how, helped to also embed reflection as a habit of lifelong learning,^46^, ^47^ ensuring that EDI was not only understood intellectually, but also embraced behaviorally.

**FIGURE 3:**
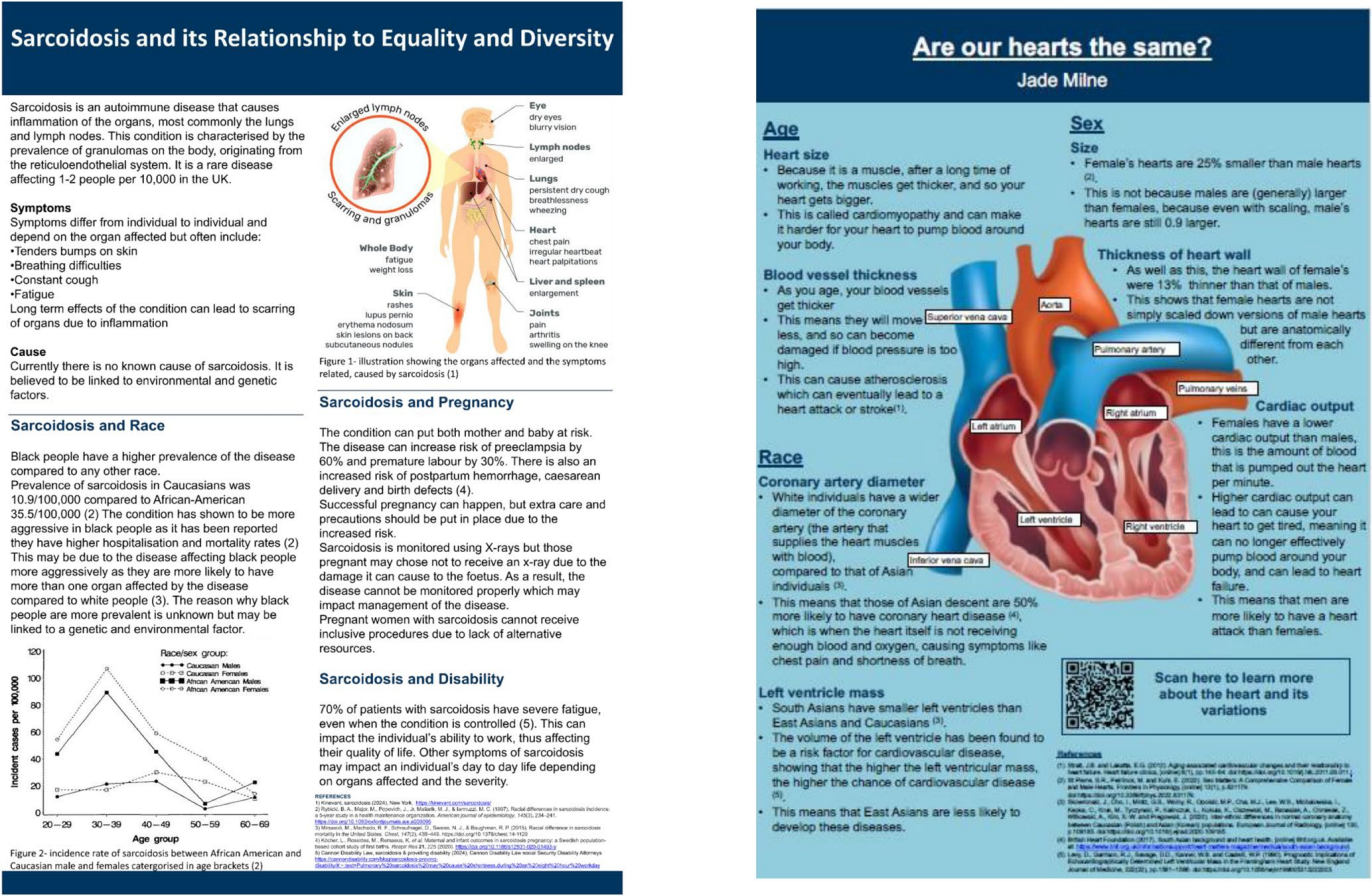
Example student work generated through the EDI workshops.

Together these workshops offer a cohesive, scaffolded approach to EDI in anatomical education, grounding what may be perceived as abstract concepts in real‐world examples and providing students with both the knowledge and tools to think critically about inclusion, representation and identity in anatomical sciences.

### Academic skills development and assessment

Building assessments directly linked with the EDI sessions was critical to give credit to the content and reflect the relevance and importance of this material. Assessments were carefully designed to align with and reinforce our program‐level learning outcomes (course information) and UofG Graduate Attributes. Students participated in formative presentations, wiki writing, and peer feedback, whereas summative in‐course components included an assessed poster, a written reflection and exam essay questions directly linked to inclusive anatomical education, ensuring that EDI was not relegated to an extracurricular concern, but embedded within the core curriculum and formal assessment. In doing so, we supported the development of key academic and transferable skills critical for success in the biosciences such as scientific writing, critical debate, oral communication, reflective thinking, and collaborative working (QAA). This approach helped students engage with EDI content while simultaneously building competencies essential for both academic progression and future employability.

### Quantitative and qualitative analysis

This project was approved by the University of Glasgow College of MVLS Ethics Committee (approval number: 200200187).

Quantitative data were collected via pre‐ and postteaching surveys using Likert‐scale^1^, ^2^, ^3^, ^4^, ^5^, ^6^, ^7^, ^8^, ^9^, ^10^ and multiple‐choice questions to evaluate students' perceptions of EDI relevance, inclusivity, and representation across anatomy, the broader life sciences curriculum and the university environment (Figure S2). Students were invited to take part in the preteaching survey by teaching staff at the start of workshop one and in the postteaching survey at the end of the last workshop. All surveys were anonymous thus pre‐ and postsurveys were not paired or linked to assessment scores. Descriptive statistics were calculated to compare perceptions before and after the EDI teaching intervention, including percentage distributions across response categories. Qualitative data were gathered through open‐ended survey responses, which were thematically analyzed using a predominantly inductive coding approach (Figure S3). Initial coding was conducted by one researcher (OV) and independently reviewed by a second researcher (MW), with final categories refined through discussion and triangulation between the two researchers to ensure reliability. This dual methods approach allowed for a rich, triangulated interpretation of the data, highlighting both measurable trends and the nuanced shifts in student understanding of EDI in anatomy.

Performance data were analyzed across consecutive cohorts from the 2021–2022 to 2024–2025 academic years. As the University of Glasgow employs a 22‐point grading scale, the average grade achieved per assessment was converted to its percentage equivalent. These converted average percentage grades were then used to calculate aggregated mean percentage performance for EDI‐linked and non‐EDI assessments. Similarly, student grades were compared for exam essays that focused on EDI‐related topics (example shown in Figure S4) compared to exam essays which did not include EDI content. All exam questions included in this analysis, including those incorporating EDI‐related content, were developed and approved through standard institutional assessment processes. These processes included internal peer review, program‐level scrutiny and external examiner oversight. EDI and non‐EDI questions were benchmarked against the same learning outcomes and academic standards and were designed to assess core anatomical knowledge, scientific reasoning and clinically or research relevant understanding. Although EDI‐related questions addressed topics such as anatomical variation and population‐level considerations, they were grounded in established biomedical and anatomical evidence rather than subjective or reflective judgment. Marking for all exam questions was conducted using the same structured marking scale with predefined criteria to ensure objectivity and consistency across markers and cohorts.

## RESULTS

A total of 139 students were eligible to participate in the surveys; response rates were 72% (*n* = 100) for the preteaching survey and 43% (*n* = 60) for the postteaching survey.

### Perceived awareness of EDI to anatomy

Students rated EDI highly relevant to anatomy teaching, with an average score of 8.5 out of 10, demonstrating an appreciation of the connection between EDI principles and anatomical education. The score was the same before (8.6/10) and after (8.5/10) the teaching intervention suggesting that students were already aware of the importance of EDI in biomedical sciences and that this was not influenced by the teaching; likely reflective of previous teaching in prehonors years at the University of Glasgow. Although students were also able to define EDI preteaching, thematic analysis of responses revealed a notable shift in their understanding of EDI. Prior to the intervention, students tended to define EDI using more foundational terms such as accessibility, equality, diversity, inclusion, representation, respect, nondiscrimination and being valued. These definitions suggested a general awareness of EDI principles, often framed in basic terminology, which also aligns with their perceived score of EDI relevance to anatomy. However, following the teaching intervention, the constructed categories reflected a deeper and more reflective engagement. Terms such as awareness, insight, transparency, life‐long learning, and no judgment became apparent, suggesting that students were beginning to view EDI not just as a static policy or solely as a moral stance, but as a dynamic, continuous and personally relevant practice. The addition of categories such as awareness, insight and life‐long particularly point to a shift toward recognizing EDI as an evolving responsibility tied to both professional development and social accountability.

Thematic analysis generated the following five themes in the student comments:

### Theme 1: Equality as sameness and fairness

Prior to the intervention, students predominantly defined equality as sameness, fairness and equal treatment. Responses emphasized identical opportunities and nondiscrimination, often without reference to structural factors, diversity or inclusion. Illustrative preintervention responses included: *Equal opportunity for everyone*, *Treating everyone the same regardless of race, religion, sex, etc*., and *Fair treatment and interactions between everyone*. While these definitions reflect well‐intentioned values, they tended to frame equality as uniform treatment rather than equitable support or contextual responsiveness.

### Theme 2: Awareness of difference and diversity

Postintervention responses demonstrated a marked increase in awareness of human difference, diversity, and protected characteristics. Students moved beyond equality as sameness toward recognizing variation across populations and its relevance to anatomy and education. Illustrative postintervention responses included*: Learning about people, their differences and how to respect them*, *Understanding anatomy being the scientific learning of how humans differ*, and *Learning and embracing the differences between people and how we can acknowledge these*. This shift reflects a transition from abstract values to disciplinary awareness, linking EDI explicitly to anatomical science.

### Theme 3: Representation and visibility in anatomy and science

Following the intervention, students increasingly emphasized representation, particularly in relation to teaching content, curriculum relevance, and scientific validity. Representation was framed as necessary for relevance, fairness, and accuracy in anatomy education. Illustrative postintervention responses included*: Ensuring that all people from different backgrounds feel represented in their studies*, *Fairness and representation of all*, and *The representation and inclusion of all individuals in our area of study without bias*. This theme was largely absent in preintervention responses, indicating a meaningful conceptual expansion.

### Theme 4: Inclusion as active practice rather than principle

Inclusion shifted from being described as a passive ideal to an active, practical responsibility. Postintervention definitions highlighted inclusion as something that must be deliberately designed, enacted, and sustained within learning environments. Illustrative responses included: *Making sure that everyone feels included and represented*, *Providing the means necessary for equal opportunity*, and *Ensuring everyone is able to participate in all aspects of the university*. This reflects a move from value statements toward applied inclusion.

### Theme 5: EDI as lifelong, ethical, and professional responsibility

A final theme constructed primarily postintervention framed EDI as an ongoing ethical commitment extending beyond the classroom and into professional and societal contexts. Illustrative responses included: *Ensuring all types of people are catered for and considered throughout life*, *A helpful life skill*, and *Crucial for awareness within an evolving society*. This theme aligns with the intended heutagogical outcomes of the intervention and indicates deeper internalization of EDI principles.

### Perceptions of inclusivity and representation

Most students evaluated body donor and prosected material in year 2 and year 3 as “somewhat inclusive” (Table 1). This remained relatively consistent across both years, even with the addition of anatomy‐focused EDI content in year 3; qualitative responses post intervention reflected a deeper and more critical engagement with the concept of inclusivity. Preteaching themes predominantly focused on visible traits such as age, sex, skin color, and race. However, postteaching responses noted the lack of ethnic and ability‐based diversity in donor materials and commented that most specimens were male while also demonstrating expanded awareness, with students identifying more complex and intersectional issues including ethnicity, disability, and the logistical limitations of the body donation program itself. Postteaching, students commonly acknowledged the reasoning for the lack of diversity in the teaching resources and noted that this lack of diversity was discussed by teaching staff (Figure 4).

**TABLE 1:** Pre‐ and postteaching students perceptions on inclusivity and representation (in %).

	Preteaching (%)	Postteaching (%)
How inclusive are the donor and prosected resources		
Not inclusive	28%	38%
Somewhat inclusive	47%	57%
Very inclusive	25%	5%
How inclusive students perceived		
Anatomy	74	52
Life sciences	76	64
University	75	60
How represented students felt in		
Anatomy	60	75
Life sciences	91	80
University	71	77

**FIGURE 4:**
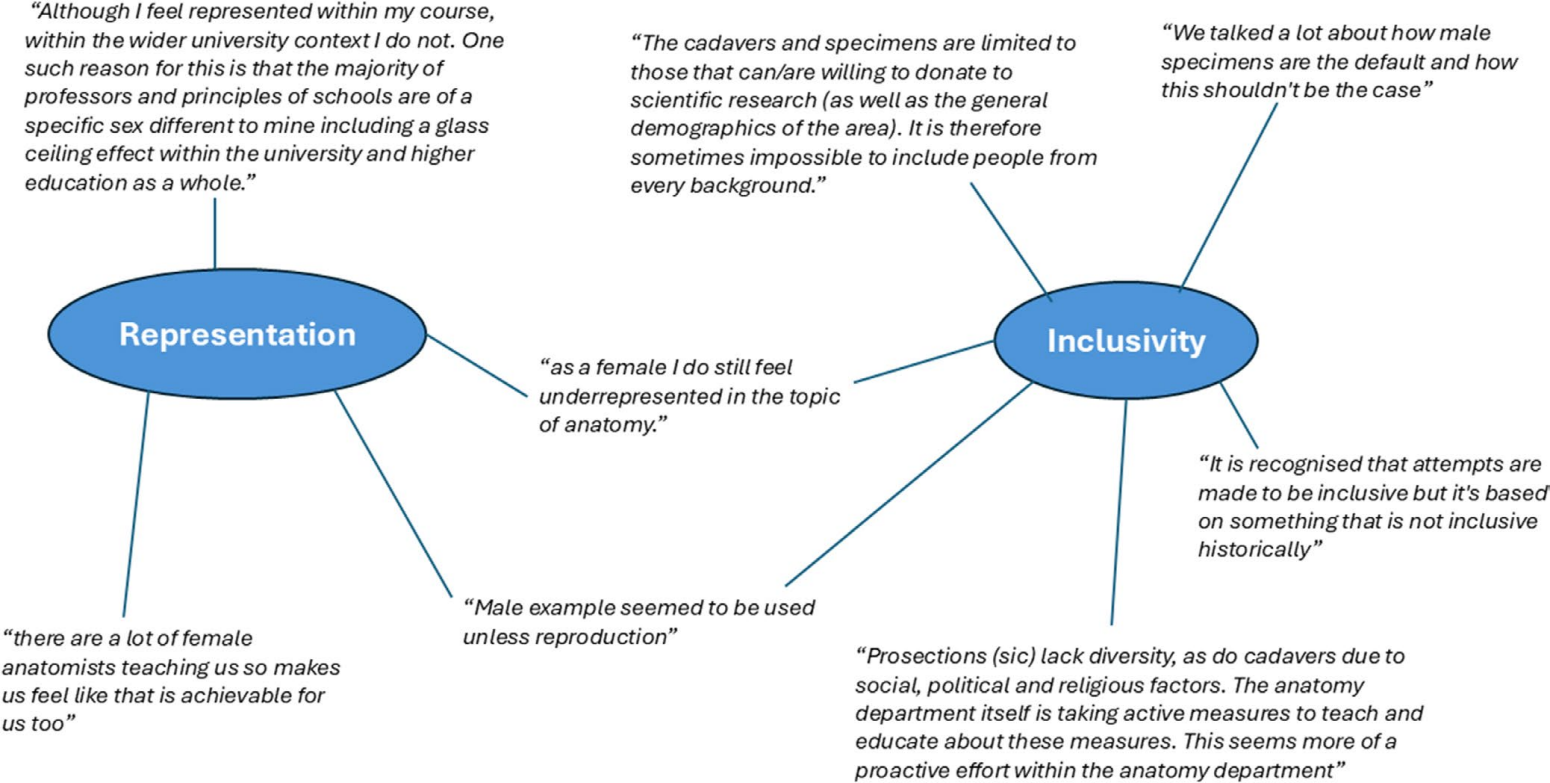
Student quotes on feelings of representation and inclusivity.

In terms of how inclusive students perceived anatomy, life sciences, and the wider university, there was downward shift likely reflecting increased awareness and critical engagement with the meaning of inclusivity rather than diminished experiences (Figure 3, Table 1). Despite this, representation within the discipline of anatomy improved postintervention, with students reporting an increase from 60% to 75% feeling “very represented.” However, this improvement was not mirrored in life sciences or university‐level perceptions, highlighting the impact of discipline‐specific interventions and role models (Table 1).

### Protected characteristics coverage

Analysis of perceived coverage of the Equality Act (2010) protected characteristics showed that sex, age, pregnancy, disability, and race were most consistently addressed in the EDI teaching within our anatomy course (Figure 5). In contrast, more social characteristics such as sexual orientation, partnerships, and religion were perceived as less well covered in the curriculum. Qualitative comments suggested that some students viewed characteristics such as marital status as less relevant in anatomy, though others recommended broader inclusion—*Some of the EDI factors have no relation to anatomy* and *Some of these characteristics wouldn't affect anatomy, such as marriage*.

**FIGURE 5:**
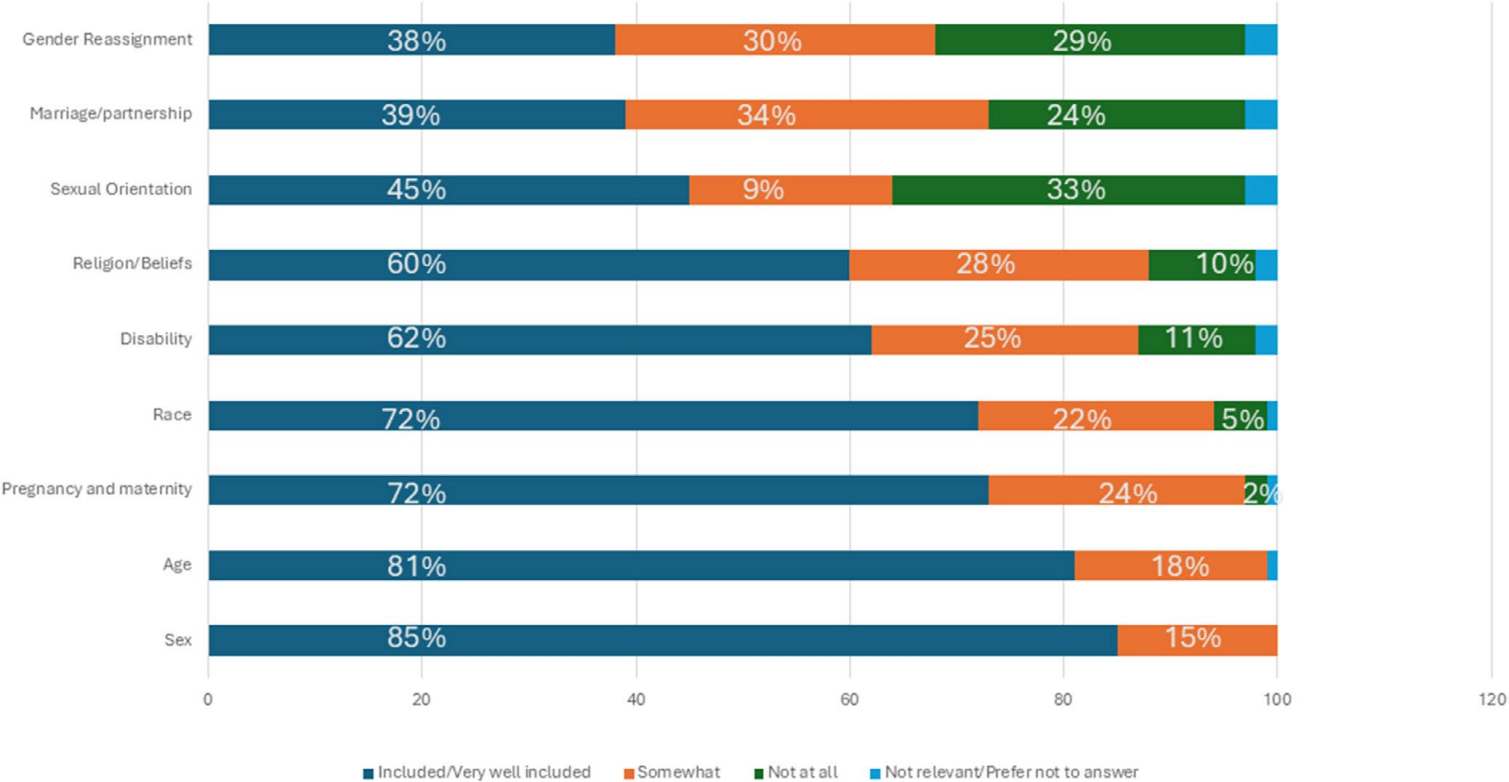
Student perceptions of the coverage of protected characteristics in EDI anatomy teaching (in %).

### Workshop evaluation

Students rated the workshops positively, with an average score of 4 out of 5. Thematic analysis of free‐text responses identified group work, personal reflection, and open discussion of EDI as particularly valuable, with many students reporting that these elements promoted critical thinking and active engagement. Quantitative coding of responses showed that 68.9% (31/45) described engagement or new perspectives/insight, whereas 15.6% (7/45) felt some sessions were overly long or had too much information and 13.3% (6/45) expressed mixed views regarding the relevance, structure, or workload of associated assessments (Figure S2).

### Assessment perceptions and learning impact

While students lacked enthusiasm about the usefulness of the EDI‐linked assessments, with just 6% of responses noting them as a useful aspect of the EDI teaching, performance data showed that across consecutive cohorts from the 2021–2022 to 2024–2025 academic years, the aggregated average percentage grades for EDI‐linked coursework were consistently higher than those for non‐EDI assessments (Figure 6). Students scored one grade point higher in exam essays that focused on EDI‐related topics (15 on a 22‐point scale which aligns to a B band, equivalent of around 68%) compared to other exam essays (14 on a 22‐point scale which aligns to a C band grade, 64%) that did not include EDI content. This trend suggests that despite lower subjective enthusiasm, the EDI assessments effectively engaged students and supported strong academic performance, reinforcing their pedagogical relevance.

**FIGURE 6:**
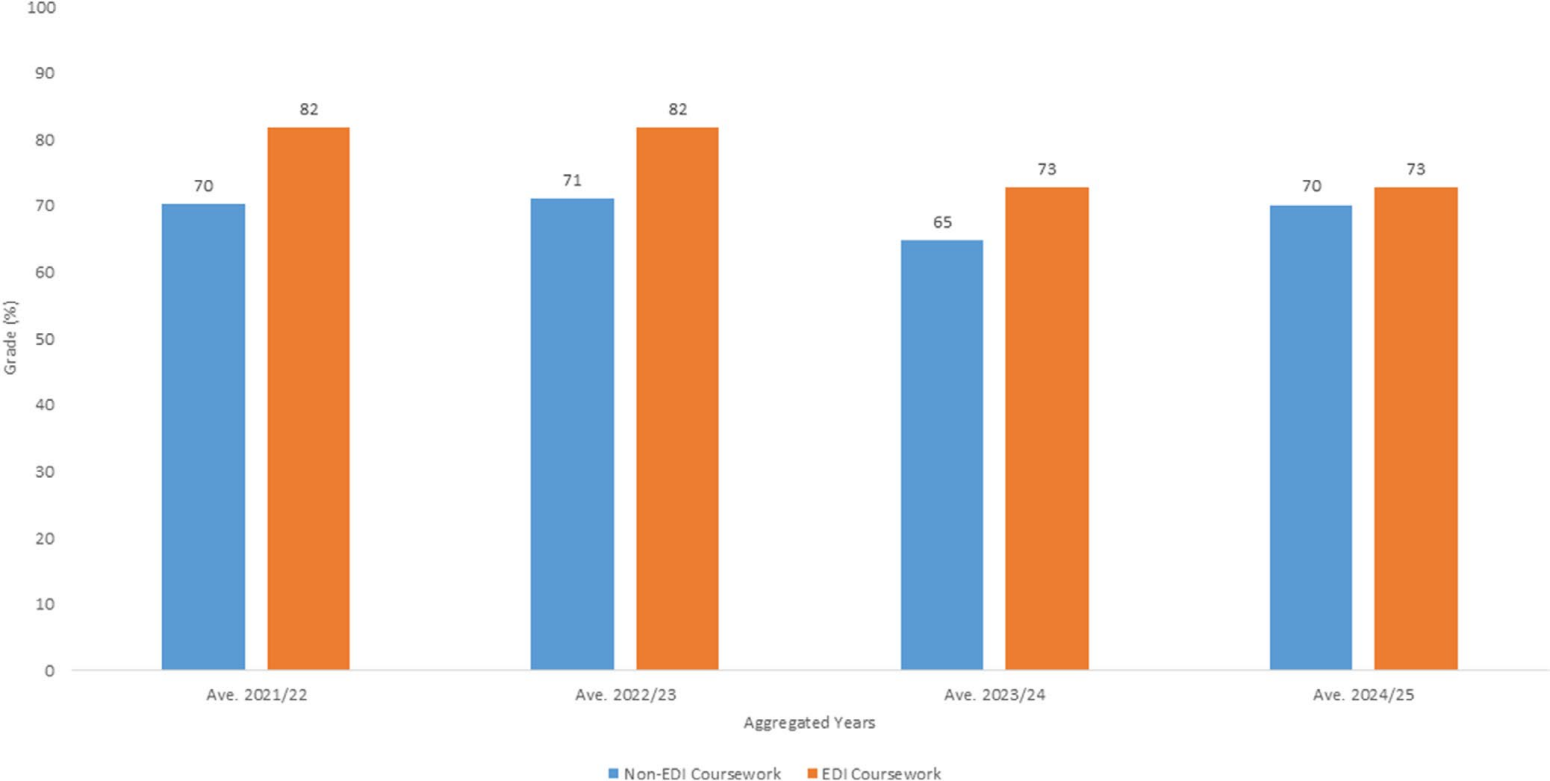
Aggregated average coursework grades (in %) for EDI‐linked and non‐EDI assessments across different student cohorts from the 2021–2022 to 2024–2025 academic years.

## DISCUSSION

### Integrating EDI as a pedagogical framework

Students welcomed the integration of EDI within year 3 of the BSc anatomy program which led to measurable and meaningful shifts in student understanding of the topic, demonstrated in their survey responses and in their academic performance. This aligns with previous research which showed that students studying anatomy agreed or strongly agreed that “improving inclusivity in anatomy education should be educator's priority”.^2^ The introduction of EDI in year 3 anatomy also had a positive impact on students' sense of representation within anatomy as a discipline. This compares favorably to the lower levels of representation noted by Longhurst and colleagues in their study of anatomy students' perceptions of EDI prior to any intervention.^2^ This impact on representation echoes other studies which reported that students were grateful for greater representation within anatomy curricula, which resulted in a greater sense of belonging in their courses.^8^

Students also rated the workshops highly and welcomed the opportunity for open discussion and reflection, identifying that this helped them to build critical thinking skills and empowered them to take ownership of their learning journey through the opportunity to select case studies, anatomists and topics that aligned with their personal values, identities, and academic interests. This again agrees with previous research that discussed the benefits of EDI when course content has relevant connections to topics of diversity and when these topics are in alignment with the intended learning outcomes or competencies.^28^ Indeed Meyer and Cui advocated for approaches similar to those used in this study, organizing students into small groups in which they discuss the factors contributing to higher risks of diseases in particular racial or ethnic groups and also discussing the ethical considerations of body donation programs.^28^ Each of the workshops described here aimed to take an intersectional approach rather than focusing on specific demographics. For example, in workshop 2, when students discussed their chosen historical anatomists, they highlighted not just an isolated characteristic such as gender or skin color but rather were prompted to consider how multiple elements of their lived experience might collectively impact on their research and any discrimination or privilege they might have faced. Similarly in workshops four and five, students were instructed to consider at least three protected characteristics in their research. This encouraged them to see how these characteristics might interact to impact the disease or anatomical structure. However, despite aiming to approach EDI through an intersectional lens, students recognized that the workshops did not equitably discuss all protected characteristics and instead focused on those more clearly aligned with the physical body. This may in part be due to the student‐centered instructional design approach in which students directed the focus of their learning and research which would naturally gravitate toward the more physical traits. This aligns with Longhurst and colleagues who showed that the majority of the anatomy students they surveyed agreed that age, disability, ethnicity, sex, and gender should all be considered in an anatomy curriculum.^2^ While the identities more obviously associated with and thus appropriate for an anatomical curriculum were commonly covered in our EDI workshops, students recognized that gender reassignment was not covered in depth during this course; this content is delivered later in the fourth year of their program. However, one student commented that gender reassignment was not relevant to anatomy; this was unexpected given the physical changes induced by gender affirming therapies, however, it was a single comment from over 60 free text comments so is unlikely to represent a wider perspective. It is important that students are encouraged to explore the full spectrum of diversity so as not to perpetuate marginalization of underrepresented populations, including a need to recognize anatomical diversity beyond all the UK protected characteristics to consider aspects like body shape.^9^ The design of these workshops should be updated to reflect this, for example, specifying that students should consider at least one of the less physical characteristics in their discussions.

### Shifting student understanding and shaping professional identity

Students shift in understanding of EDI was evident in their definitions of EDI; while they recognized its relevance to anatomy prior to the intervention, their postteaching responses revealed a more critical and nuanced conceptualisation of EDI, indicating a shift from surface‐level awareness to deeper, reflective engagement. Such development reflects elements of transformative learning theory, which emphasizes personal perspective change through critical reflection and experiential engagement,^48^ and aligns with broader findings on EDI teaching as a catalyst for professional growth.^24^ Previous studies have already demonstrated that professional growth and identity are shaped by experiences that encourage students to see themselves as professionals who embody humanistic values, ethical awareness, and compassionate practice with anatomy teaching offering opportunities for this through formal teaching with donor bodies but also through informal influences such as the hidden curriculum and learning environment.^25^, ^49^, ^50^, ^51^, ^52^, ^53^ It is therefore unsurprising that these EDI anatomy workshops can also shape student growth and identity. Similarly, overall ratings of inclusivity in anatomy also remained consistent before and after the teaching intervention. As their understanding of EDI deepened, their postteaching reflections on inclusivity revealed a more nuanced awareness of the limitations in the diversity of body donations and anatomical or medical imagery. A further consideration relates to the ethical, cultural, and historical dimensions of donor practice. Although our workshops prompted students to reflect on donor demographics, future iterations for healthcare practitioners could engage more explicitly with the concept of donors as *first patients* or *silent mentors*, an approach shown to foster professionalism, ethical awareness and deeper respect for the individuals who enable anatomical learning.^54^, ^55^, ^56^ This was not immediately relevant for this study cohort of science students but would be an important consideration for medical profession students.

Students also reported an improved sense of representation within anatomy after the teaching, but a similar increase was not seen in their sense of representation within the broader life sciences curriculum or the university as a whole. This is likely to be attributed to the discipline‐specific nature of the EDI content introduced, by explicitly anchoring EDI discussions in the students' core anatomical subject area, the teaching created relevance and resonance, fostering a deep sense of identity with the materials. Moreover, by establishing open and inclusive learning environments and enabling students to critically explore identity and representation through anatomy, the intervention actively cultivated belonging and a sense of community that are key in enhancing student engagement, particularly important for those from minoritized backgrounds.^57^ The deliberate alignment of EDI with students' academic/professional identities may therefore have played a role in enhancing their connection to the subject and to each other.

### Academic skills, knowledge, and performance

The EDI content developed provided the students with the foundational knowledge required to engage with complex EDI topics, while the workshop series were formatted to take the students from a more independent pedagogical approach to a more heutagogical approach that encouraged greater learner autonomy.^46^, ^47^ This student‐centered instructional design supported self‐determined learning, a core tenet of heutagogy,^46^, ^47^ and enabled students to engage more critically and authentically with the material which will hopefully extend beyond the class and into their lifelong learning and future professional contexts. This aligns with the UDL guidelines^38^ with the workshops encouraging students to develop an awareness of self and others (9.2), promoting reflection (9.3), cultivating empathy (9.4), addressing biases in the use of languages and symbols and in modes of expression and communication (2.4 and 5.4) and challenging exclusionary practices (6.5). Such approaches are associated with increased learner motivation and personal relevance which enhance both engagement and knowledge retention.^58^, ^59^ This approach was one previously highlighted as important to effect change in students and improve inclusion.^9^ This was demonstrated in our study with students engaging well with the sessions and consistently achieving higher grades in EDI coursework and exam essays compared to non‐EDI assessments. It should be acknowledged that EDI is more of a social construct which students might find easier to learn and explain compared to the detailed anatomical knowledge assessed in their other assessments. However, given that EDI‐related questions were designed to assess anatomical knowledge and applied scientific reasoning and were graded using the same objective standards as all other assessments, the difference observed over 4 years is more plausibly explained by variation in topic difficulty rather than increased subjectivity in marking. This might in part explain the higher averaged grades for the EDI assessments compared to the other anatomy assessments. However, the improved grades may also be attributed to the iterative nature of the teaching intervention, which scaffolded students' engagement with EDI concepts across multiple workshops, allowing for progressive skill development and deeper conceptual integration. Furthermore, the content was embedded directly into their curriculum, intertwined and aligning with their more traditional anatomical teaching therefore encouraging the students to see this as part of their core curriculum rather than additional or optional content as may be seen in other disciplines.^13^, ^60^ The benefits of integrating EDI within the course content and aligned with the intended learning outcomes or competencies had been previously highlighted.^28^ This constructive alignment of learning outcomes, activities and assessments created a coherent pedagogical trajectory that enabled students to build academic confidence while applying inclusive thinking to disciplinary content. Constructive alignment not only supports effective learning, but also enhances learner autonomy and academic performance by making expectations transparent and meaningfully connected to students learning journeys.^61^ By embedding EDI throughout both formative and summative tasks and encouraging repeated reflection and higher‐order synthesis, the intervention enabled students to strengthen critical academic competencies that underpinned their academic achievement. This aligns with the idea of taking EDI beyond teaching and integrating it into assessment approaches too^9^ This reinforces the argument that EDI is not merely an ethical or values‐based addition to the curriculum, but a rigorous intellectual endeavor that supports educational excellence and graduate preparedness. However, despite this approach, our students failed to see the usefulness of the EDI‐linked assessments, with just 6% of responses noting them as a useful aspect of the EDI teaching. This could reflect student apathy around assessments generally or perhaps we did not pick an assessment style which encouraged students to best apply and consolidate their knowledge. The authors tried to accommodate different student preferences by including a poster and reflection as in course assessments as well as exam style questions embedded in their final year exam. However, alternative assessment types could be explored with students as partners to design assessments which better apply their learning, considering more modern discussive approaches like students creating a podcast which would allow them to include more discussion and reflection as well as demonstrating their anatomical knowledge.

### Areas for future work

The survey data here were self‐reported, which could have introduced some potential response biases. While the combination of quantitative and qualitative data offered valuable insights into the overall impact of the intervention, the fully anonymous nature of data collection prevented the tracking of individual students' learning journey across time. As a result, we were unable to capture the nuanced evolution of individual understanding or assess the specific impact the EDI teaching had on each student, limiting the depth of analysis regarding personal transformation. Future research could address these limitations by incorporating coded, linked pre‐ and postintervention data to capture individual learning trajectories more effectively. It would also be interesting to follow up this cohort postgraduation to see how their reflections evolve after entering the workforce and if their field of work has any additional impact on this. Additionally, adapting this pedagogical framework to other disciplines and international contexts could enhance the transferability of findings and broaden its impact across higher education.

Although this research adopted a longitudinal study design, it was conducted on a single UK‐based institution focusing on one biosciences disciplinary cohort, which may limit the findings' generalisability. The cohort did not fully represent the degree of diversity either in the UK or globally, with the majority of students being White Scottish females. Further studies are needed applying a similar framework and teaching approaches in different institutions globally to expand this representation. The workshops could be used as is with other anatomical sciences programmes globally to explore how students from more diverse backgrounds compare in their responses to the same materials. Alternatively, the content from the workshops could be adapted to be incorporated into existing anatomy labs or problem‐based learning sessions in medical curricula for example. While this intervention was designed within a UK anatomical sciences context, several elements of the framework are broadly transferable. Components closely tied to local structures—such as the demographic profile of UK body donors, UK‐based legislative framing through the Equality Act 2010, and the cultural expectations of a Scottish higher‐education environment—would need modification when applied elsewhere. However, the core pedagogical design principles underpinning the framework are widely applicable across disciplines and geographical contexts. These include the use of scaffolded, constructivist workshop sequences; EDI discussions anchored explicitly in disciplinary content; authentic assessments that reward critical reflection; and the deliberate creation of psychologically safe learning environments that encourage open dialogue. For programs with more diverse student cohorts or different cultural contexts, co‐creation approaches such as inviting students to contribute examples, case studies, or lived experiences reflective of their identities may enhance both cultural relevance and student ownership of learning.

The framework is easily adaptable for medical curricula and health‐professional programmes, where the relevance of EDI‐grounded anatomical variation may be even more pronounced. For example, sessions on diagnostic bias, population‐specific disease presentations, or the clinical implications of anatomical diversity could be embedded within problem‐based learning or clinical‐skills teaching. In international or culturally diverse institutions, these workshops could be tailored by integrating local donor demographics, region‐specific health inequities, or historical narratives relevant to the local context. Adjustments may also be required to ensure cultural and linguistic appropriateness, particularly in settings where concepts such as intersectionality or protected characteristics are framed differently or have no direct legislative equivalents. Additionally, the framework could be used to develop content for other subjects such as physiology or engineering, designing workshops that focus on the diversity of historical figures and practices, the inclusivity of application of knowledge in the field, and training students to recognize underlying bias and challenge those. In this way, the framework offers a flexible pedagogical model that retains its core principles while allowing meaningful localization to different curricula, cultural settings and learner populations.

Although the intervention fostered an inclusive and psychologically safe learning environment, we recognize that it did not explicitly address cultural safety for Indigenous or other marginalized learners. Given that anatomical education intersects with complex cultural and historical relationships to the body, including legacies of colonial exploitation in the acquisition and use of anatomical material, future iterations of this framework could incorporate more locally relevant culturally grounded discussions and ensure that students from diverse backgrounds feel that their histories, identities, and perspectives are acknowledged and respected.

### Wider implications for curriculum reform

This intervention offered a replicable and transferable framework for embedding intersectional EDI content into higher education in ways that are pedagogically informed and student‐centered, aligned with sectorwide expectations such as the QAA and AdvanceHE. More broadly, this work highlighted that EDI curriculum design should be horizontally embedded across multiple learning experiences, not only for individual courses but also at program level and scaffolded to allow progressive development of conceptual understanding. Encouraging institutions to move beyond compliance models of EDI toward transformative, co‐constructed learning experiences is vital to support diverse learners and prepare graduates who knowledgeable, socially and culturally aware. This study demonstrated that small, consistent changes can make a big impact, in agreement with the data collected by Evans and colleagues.^8^

## CONCLUSION

The scaffolded learning design, underpinned by thematic anchors and grounded in social constructivism, enabled students to progressively deepen their EDI understanding from historical critique to structural exploration and real‐world applications. This pedagogical intervention reframed anatomy not as a fixed or purely technical discipline, but as a dynamic and dialogic space for interrogating identity, diversity, inclusion, and representation. Rather than treating EDI as abstract theory, the workshops related it directly to their discipline and fostered critical thinking, reflective dialogue and peer‐led inquiry. Ultimately, this integration of EDI into year 3 of the BSc Anatomy program supported students not only in becoming knowledgeable anatomists but also equity‐conscious professionals equipped to navigate diverse academic/professional settings. Importantly, this approach was also linked with improved academic performance in the field. Building on these findings, this framework offers a scalable model that can be adapted across disciplines, institutions, and sectors to meaningfully embed intersectional EDI in ways that enhance belonging, critical thinking, and academic success.

## AUTHOR CONTRIBUTIONS

**Michelle Welsh:** Conceptualization; investigation; writing – original draft; methodology; writing – review and editing; formal analysis; project administration; data curation; resources; supervision. **Ourania Varsou:** Investigation; writing – original draft; methodology; writing – review and editing; formal analysis; data curation; supervision; resources.

## FUNDING INFORMATION

No funding was received for the work undertaken in this study.

## CONFLICT OF INTEREST STATEMENT

The authors have no conflict of interest to declare.

## ETHICS STATEMENT

This project was approved by the University of Glasgow College of MVLS Ethics Committee (approval number: 200200187).

## Supporting information


**Supplementary Figure 1.** Code of conduct for EDI anatomy workshops at the University of Glasgow.
**Supplementary Figure 2**. Pre‐ and postteaching surveys.
**Supplementary Figure 3**. Workshop evaluation comments presented as the proportion of responses for each statement or theme.
**Supplementary Figure 4**. Example of EDI‐focused exam question and suggested answer.

## Data Availability

The data that support the findings of this study are available from the corresponding author upon reasonable request.
