# Supplementary material for: Seeing ourselves in anatomy: Embedding equity, diversity, and inclusivity to transform learning in a UK BSc curriculum
Source: Anat Sci Educ. 2026 Aug 27;19(11):1981–97. doi: 10.1002/ase.70333 (PMC13736539; doi:10.1002/ase.70333)
Supplement: Supplementary file 1 [file ASE-19-1981-s001.docx]

**^[1]^^[2]^Title Page**

**Title**: Seeing Ourselves in Anatomy: Embedding Equality, Diversity and Inclusion to Transform Learning in a UK BSc Curriculum

**Short Title:** Embedding EDI in Anatomy

**Keywords**: Equality, diversity and inclusion; anatomical education; intersectionality; resource diversity; scaffolded constructivist pedagogy

**Authors & Affiliations**:

Michelle Welsh^1*^ and Ourania Varsou^1^

1. Anatomy Facility, School of Medicine, Dentistry and Nursing, College of Medical, Veterinary and Life Sciences, University of Glasgow, United Kingdom.

**ORCID IDs:**

Michelle Welsh: <https://orcid.org/0000-0003-1469-0201>

Ourania Varsou: <https://orcid.org/0000-0002-7789-1185>

***Corresponding Author:**

Dr Michelle Welsh,

Senior Lecturer

Deputy Head of Anatomy (Learning and Teaching)

School of Medicine, Dental and Nursing

College of Medical, Veterinary and Life Sciences

Room 342

Thomson Building

University of Glasgow

Glasgow

G12 8QQ

Tel: 0141 330 5926

Email: [Michelle.Welsh@glasgow.ac.uk](mailto:Michelle.Welsh@glasgow.ac.uk)

**Ethics Statement:** This project was approved by the University of Glasgow College of MVLS Ethics Committee (approval number: 200200187).

**Supplementary File**

**Supplementary Figure 1: Code of conduct for EDI anatomy workshops at the University of Glasgow**

**Pre- and Post-Teaching Survey Questions**

1. Did you take the Contemporary Issues in Biology 2 (Biology 2Y) course? Yes/No
2. How would you define Equality, Diversity and Inclusion? Open text
3. Is Equality, Diversity and Inclusion relevant to the teaching of human anatomy? Answered on a 1-10 scale with a ‘prefer not to answer’ option and clarification that 1=no and 10=yes
   a. Option to provide explanation of answer.
4. Do you think the human anatomy cadavers/prosected material used in teaching (for example in the 2C labs in year 2) at the University of Glasgow is diverse and inclusive? Answered on a 1-10 scale with a ‘prefer not to answer’ option and clarification that 1=no and 10=yes
   a. Option to provide explanation of answer.
5. How inclusive do you feel anatomy is as a discipline/topic (generally, rather than as a university course)? Answered on a 1-10 scale with a ‘prefer not to answer’ option and clarification that 1=not inclusive and 10=very inclusive
   a. Option to provide explanation of answer.
6. How inclusive do you feel the School of Life Sciences is? Answered on a 1-10 scale with a ‘prefer not to answer’ option and clarification that 1= not inclusive and 10= very inclusive
   a. Option to provide explanation of answer.
7. How inclusive do you feel is the University of Glasgow? Answered on a 1-10 scale with a ‘prefer not to answer’ option and clarification that 1=not inclusive and 10= very inclusive
   a. Option to provide explanation of answer.
8. How well represented do you feel in anatomy as a discipline/topic (generally, rather than as a university course)? Answered on a 1-10 scale with a ‘prefer not to answer’ option and clarification that 1=not represented and 10=very represented
   a. Option to provide explanation of answer.
9. How well represented do you feel in the School of Life Sciences? Answered on a 1-10 scale with a ‘prefer not to answer’ option and clarification that 1=not represented and 10=very represented
   b. Option to provide explanation of answer.
10. How well represented do you feel at the University of Glasgow? Answered on a 1-10 scale with a ‘prefer not to answer’ option and clarification that 1=not represented and 10=very represented
    c. Option to provide explanation of answer.
11. Do you have an academic role model (For example, a person who has inspired you to pursue your studies/career)? If so, please describe them. Open text

**Pre- Teaching Survey Question Only**

1. How well do you feel *insert protected characteristic* is considered in anatomy/human biology teaching in years 1 and 2? (For example, in the teaching of the cardiovascular system, were age- or gender-related differences considered?) Answered on a 1-10 scale with a ‘prefer not to answer’ option and clarification that 1=not considered and 10=well considered
   a. Option to provide explanation of answer.
      i. age
      ii. gender reassignment
      iii. marriage and civil partnership
      iv. pregnancy and maternity
      v. disability
      vi. race including ethnic or national origin
      vii. religion or belief
      viii. sex
      ix. sexual orientation

**Post-Teaching Survey Questions Only**

1. How well do you feel *insert protected characteristic* is considered in anatomy/human biology teaching in year 3? (For example, in the teaching of the cardiovascular system, were age- or gender-related differences considered?) Answered on a 1-10 scale with a ‘prefer not to answer’ option and clarification that 1=not considered and 10=well considered
   a. Option to provide explanation of answer.
      i. age
      ii. gender reassignment
      iii. marriage and civil partnership
      iv. pregnancy and maternity
      v. disability
      vi. race including ethnic or national origin
      vii. religion or belief
      viii. sex
      ix. sexual orientation
2. How would you rate the overall quality of the EDI workshops? Answered on a 1-10 scale with clarification that 1=low quality and 10=very high quality
3. Which part(s) of the equality, diversity and inclusion workshop did you find the most useful? (Please select as many as applicable)
   a. The specific topics covered
   b. The opportunity to discuss equality, diversity and inclusion as a concept
   c. The opportunity to reflect on my own opinions and experience of equality, diversity and inclusion
   d. The opportunity to develop my group working skills
   e. The assessments associated with these workshops
   f. Other (please, state…)
4. What did you like the most about the equality, diversity and inclusion workshop? Open text
5. Was there anything you disliked or think we can improve in the teaching of equality, diversity and inclusion in Anatomy 3? Open text
6. Please feel free to add any additional comments below. Thank you for your feedback. Open text

**Supplementary Figure 2: Pre- and post-teaching surveys**

| **Workshop Component students found useful** |  |
| --- | --- |
| The opportunity to develop my group working skills | **26%** |
| The opportunity to reflect on my own opinions and experience | **24%** |
| The opportunity to discuss EDI as a concept | **23%** |
| The specific topics covered | **20%** |
| The assessments associated with these workshops | **6%** |
| Other | **0.4%** |
|  |  |
| **Workshop Component students liked** |  |
| Exposure to new perspectives and open discussion (e.g. diverse views, reflection, debate) | **34%** |
| Content and topics covered (e.g. anatomy and EDI, protected characteristics, assessments) | **29%** |
| Group work and peer collaboration | **14%** |
| Personal learning and increased awareness (e.g. reflection, self-awareness, new insight) | **11%** |
| Connections to science, anatomy, and real-world issues | **9%** |
| Delivery format (e.g. online, menti polls, clarity) | **3%** |
|  |  |
| **Workshop Component students disliked or would like to improve** |  |
| Workshop structure and delivery (too long, too passive, better balance with other sessions) | **22%** |
| Assessment concerns (less summative, reduce weighting, better timing) | **17%** |
| Better integration with anatomy content (apply EDI to anatomical differences) | **15%** |
| More inclusive or specific content (e.g. gender identity, broader protected characteristics) | **15%** |
| Organisation of HKU collaboration (confusing, poorly timed, unclear expectations) | **12%** |
| Desire for more examples and practical relevance | **7%** |
| Positive feedback / no improvements suggested | **7%** |
| Preference for in-person or on-campus delivery | **5%** |

**Supplementary Figure 3: Workshop evaluation comments presented as the proportion of responses for each statement or theme.**

A. Using the anatomical illustration of a nephron as a guide (Figure 4), explain the anatomy of the following components of a nephron: renal corpuscle, loop of Henle, proximal convoluted tubule, distal convoluted tubule and collecting duct. Discuss the function(s) for each of these structures. *(40% of the marks for this question)*
B. The prevalence of kidney failure is five times higher in individuals from minority ethnic groups. The specific reasons for this increased risk are not completely understood. However, it is suspected that this could be partially attributed to the higher instances of diabetes and high blood pressure within these minority ethnic groups. These conditions are the leading causes of kidney failure. Using your knowledge of equality, diversity and inclusion discuss **TWO** reasons as to why it is important to better understand the high prevalence of kidney failure in minority ethnic groups. *(30% of the marks for this question)*
C. You have been given funding to investigate the high prevalence of kidney failure in minority ethnic groups. Design an experiment to investigate this. In doing so, include details such as your suggested study population, variables you intend to measure or analyse and the overall design of your study. Students are **NOT** required to explain detailed morphology in their answer. Ensure you make your study relevant to minority ethnic groups. *(30% of the marks for this question)*

Example answers for part B might include –

- Personalised healthcare tailored to the needs of these specific minority ethnic groups leading to improved diagnosis, prognosis, etc.
- Health equity by identifying and addressing the unique health challenges faced by minority ethnic groups, ensuring that all communities have access to appropriate and effective healthcare.
- Public health policies that specifically address the needs and risks of these populations for improved prevention, screening, etc.
- Prevention strategies tailored to the needs of these populations including lifestyle and diet modifications, regular health screenings and community health education.
- Research and development by understanding the causal factors in this specific minority ethnic groups that can inform future healthcare.

Example answer **for part C might include -**

Study population:

- Select a diverse sample of individuals from various minority ethnic groups, ensuring representation across different demographics such as age, gender, socioeconomic status, and geographical location.
- Include individuals with diagnosed kidney disease as well as those without a history of kidney-related conditions for comparative analysis.
- Top answers will discuss both a diverse group and patients with kidney disease.

Variables:

- Gather demographic data including ethnicity, age, gender, socioeconomic status, educational level, and access to healthcare. Answers do not have to list all these factors.
- Assess medical history, including prevalence of diabetes, high blood pressure, obesity, family history of kidney disease, and other relevant comorbidities. Answers do not have to list all these factors.
- Measure biochemical parameters such as blood glucose levels, blood pressure, serum creatinine, estimated glomerular filtration rate (eGFR), urinary protein levels, and lipid profiles. Answers do not have to list all these factors.
- Top answers will discuss collection of demographics, medical information and biochemical parameters with some examples for each.

Study design (one of the following)

- Employ a mixed-methods approach incorporating both quantitative and qualitative research methods.
- Conduct surveys, interviews, and focus group discussions to gather insights into cultural, social, and behavioural factors influencing kidney health in minority ethnic groups.
- Utilise medical records and laboratory data for quantitative analysis to assess the prevalence of kidney disease and associated risk factors.
- Implement longitudinal follow-up to track disease progression and assess the effectiveness of interventions over time.
- Collaborate with community organisations, healthcare providers, and policymakers to ensure community engagement, cultural sensitivity, and relevance of the study findings.

Top answers will show flow and cohesion of ideas between the study design, variables and study population.

**Supplementary Figure 4: Example of EDI-focused exam question and suggested answer.**
